# Inter-Rater Agreement Between a Trained Nurse and Physicians in FAST Examination of Trauma Patients: A Pilot Study in the Emergency Department

**DOI:** 10.3390/healthcare14091152

**Published:** 2026-04-25

**Authors:** Meropi Mpouzika, George Athinis, Maria Karanikola, Stelios Parissopoulos, Georgios Papageorgiou, Christos Rossis, Evangelia Giannelou

**Affiliations:** 1 Department of Nursing, School of Health Sciences, Cyprus University of Technology, Limassol 3036, Cyprus; maria.karanikola@cut.ac.cy (M.K.); grgs_15@msn.com (G.P.); c.rossis1998@gmail.com (C.R.); 2 Emergency Department, Nicosia General Hospital, Nicosia 2031, Cyprus; george.athinis@hotmail.com; 3 Department of Nursing, University of West Attica, 12243 Athens, Greece; spariss@uniwa.gr; 4 Department of Haematology, University of Cambridge, Cambridge CB2 0AW, UK; eg722@cam.ac.uk

**Keywords:** diagnostic accuracy, diagnostic ultrasound, emergency department, emergency nurse, emergency ward, focused assessment with sonography in trauma (FAST), inter-rater agreement, nurse-performed ultrasound, point-of-care ultrasound (POCUS), trauma

## Abstract

**Background/Objectives**: Trauma management in emergency departments (EDs) requires rapid and reliable diagnostic tools. The Focused Assessment with Sonography in Trauma (FAST) is a bedside ultrasound examination used for the early detection of free fluid in the intraperitoneal cavity, pericardium, and pleural spaces. Expanding FAST use to trained emergency nurses may support timely bedside evaluation in high-demand settings. However, data on agreement with physicians remains limited. This study aimed to evaluate the inter-rater agreement between a trained emergency nurse and physicians in performing FAST and to explore the diagnostic accuracy of nurse-performed FAST compared with computed tomography (CT). **Methods**: A prospective pilot observational agreement study was conducted between October and December 2023 in the ED of a general hospital in Cyprus. FAST examinations were independently performed by a nurse trained in FAST and by physicians from the radiology department. Four anatomical areas were assessed: right upper quadrant (RUQ), left upper quadrant (LUQ), subxiphoid-pericardial area (SUPH), and suprapubic area (BLADDER). Findings were recorded independently to promote blinding. Diagnostic performance of nurse-performed FAST was explored in a subset of patients undergoing CT. **Results**: The sample included 68 trauma patients, of whom 58 underwent FAST by both the nurse and the radiologists and were included in the inter-rater agreement analysis. Fluid was detected in four patients (6.9%) in the RUQ area and in one patient (1.7%) in both the LUQ and SUPH regions, while no positive findings were recorded in the BLADDER area. Agreement in the RUQ area was 98.3% (Cohen’s kappa = 0.85, *p* < 0.001) while agreement was observed in all cases in the SUPH region (100%, Cohen’s kappa = 1.00, *p* < 0.001), although this finding was based on a single positive case. High observed agreement was also noted in LUQ (98.3%) and BLADDER regions; however, Cohen’s kappa could not be reliably estimated in these regions due to limited variability and the very small number of positive cases. In a subgroup of patients who underwent CT (n = 23), as well as in an additional Trauma Team subgroup (n = 10), diagnostic accuracy estimates were 100% for sensitivity and specificity; however, these estimates were based on a very small number of positive cases (only two positive cases in each subgroup) and were associated with wide confidence intervals. **Conclusions**: This pilot study suggests that, under specific training conditions, a trained emergency nurse may achieve a high level of agreement with physician assessments when performing FAST. The findings regarding diagnostic accuracy are preliminary and should be interpreted with caution due to the small sample size and low number of positive cases. Further studies with larger samples and multiple operators are required to confirm these findings and to evaluate their clinical implications. Future research is also needed to determine whether nurse-performed FAST may contribute to improved patient safety and emergency department workflow.

## 1. Introduction

Trauma is one of the most common reasons for patients to visit the Emergency Department (ED) and is associated with increased morbidity and mortality rates. Timely and accurate identification of trauma-related injuries is critical for improving patient outcomes, particularly in cases of haemodynamic instability. Delayed diagnosis of intra-abdominal or pericardial effusion can lead to rapid deterioration of the patient and increased risk of mortality [1].

In abdominal and chest trauma, computed tomography (CT) and bedside ultrasound (Point-of-Care Ultrasound, POCUS) are the basic non-invasive imaging tools used in emergency medicine to identify injuries [2]. Although CT is the reference standard for assessing trauma patients, the need to transport often hemodynamically unstable patients, exposure to ionizing radiation, the duration of the examination and its cost limit its use as a first-line method. In contrast, POCUS can be performed immediately in the resuscitation area, simultaneously with the provision of care, contributing to rapid clinical decision-making [2,3].

The use of ultrasound to detect free fluid in trauma patients began in the 1980s in Europe by surgeons, and Focused Assessment with Sonography in Trauma (FAST) was established as a POCUS examination for detecting free fluid in trauma patients in the 1990s [4]. FAST is used to assess the presence of free fluid in the intraperitoneal cavity, pericardium and pleural spaces [5] and is incorporated into the Advanced Trauma Life Support (ATLS) algorithm [6] as a rapid, non-invasive, and radiation-free tool in trauma assessment [7]. However, the literature highlights significant variation in the sensitivity, specificity, positive and negative predictive values of FAST [8], making it necessary, in selected cases, to perform a supplementary CT [9].

FAST examination is considered a standard of care in the initial evaluation of trauma patients [7] and has been shown to be effective when performed rapidly, provided that standardized training and practice programs are implemented [8,10,11,12]. Traditionally, FAST examinations are performed by physicians. However, the American College of Emergency Physicians recommends that FAST may also be performed by other healthcare professionals, including emergency nurses, particularly in the ED, following appropriate training [13].

Studies evaluating the ability of non-physicians to perform FAST are limited [13,14,15,16], and the available data on nurses working in the ED are relatively scarce [17,18]. Furthermore, existing evidence is heterogeneous, with variability in study design, training approaches, and reference standards [17,18]. The literature has primarily focused on diagnostic performance and feasibility, while less emphasis has been placed on inter-rater agreement between nurses and physicians, especially using independently performed assessments within the same clinical setting.

In this context, the aim of this study was to evaluate the inter-rater agreement between a trained emergency care nurse and physicians in performing and interpreting FAST examinations in trauma patients in the ED. A secondary, exploratory objective was to assess the diagnostic accuracy of FAST performed by the nurse by comparison with CT findings.

This study was conceptually guided by the assumption that a trained emergency care nurse could achieve a high level of agreement with physicians and demonstrate acceptable diagnostic performance when performing and interpreting FAST examinations.

## 2. Materials and Methods

### 2.1. Design and Setting

This is a pilot, prospective observational agreement study conducted at the ED of Nicosia General Hospital, the largest hospital in Cyprus. This ED, with a capacity of 23 beds, serves approximately 150–200 patients daily and has a Trauma Team. This Trauma Team was created based on the guidelines of the American College of Surgeons [19], adapted to the environment of Cyprus, and includes a specialist surgeon, two trainee surgeons and three nurses from the ED. The Trauma Team is activated after notification from the ambulance transporting the injured patient, based on specific criteria (see below for participant inclusion criteria). Convenience sampling was employed and included patients who met the inclusion criteria, during the periods when the nurse-researcher was available at the ED. Given the pilot nature of the study, no formal sample size calculation was performed. The sample size was based on feasibility and the number of eligible patients presenting during the study period. The study was conducted between 1 October and 31 December 2023.

### 2.2. Participants

#### Study Inclusion Criteria

The study sample consisted of injured patients who attended the ED and met the following criteria:

- Adults (>18 years old);
- Presence of injury mechanism resulting from a fall, trauma, injury, or traffic accident;
- A referral for FAST was issued by the ED physician;
- A referral for FAST was issued by the Trauma Team in the following cases [19]:
  ✓ Confirmed systolic blood pressure < 90 mmHg;
  ✓ Penetrating gunshot wounds to the neck, chest or abdomen;
  ✓ Patients who have been intubated in the field and are being transported directly to the trauma center;
  ✓ Patients with respiratory distress or need for an emergency airway;
  ✓ Patients transferred from another hospital with ongoing respiratory distress (except those already intubated and respiratory stable);
  ✓ Glasgow Coma Scale < 9 with injury mechanism due to trauma;
  ✓ Transport of a patient from another hospital who needs continuous blood transfusion;
  ✓ At the discretion of the emergency physician.
- Signed consent from the patient or their relatives.

Exclusion criteria were:

- Life-threatening conditions upon patient arrival, such as overt major external bleeding, clinically apparent traumatic brain injury, and gastrointestinal bleeding (to avoid delaying patient management);
- Patients with a high body mass index > 40 and pregnant women (due to the difficulty of obtaining accurate images);
- Not knowing Greek or English (due to not understanding the consent form).

### 2.3. Ultrasound Equipment and Data Recording

The data collection tool used in the study was the Butterfly iQ+ portable ultrasound device (Butterfly Network Inc., Guilford, CT, USA). This is a portable device consisting of an ultrasound probe that connects to the screen of a smartphone or tablet. The images from the ultrasound scan were stored in a secure cloud environment accessible only to the device owner.

#### Description of the FAST Protocol

- FAST is performed by placing the ultrasound probe in four predetermined ultrasound windows, with the aim of rapidly detecting free fluid in the intraperitoneal, pericardial cavity, and pleural spaces typically within 5 min.
- The windows include:
  ✓ Abdomen right upper quadrant (RUQ): examination of the hepatocolic angle (Morrison’s pouch) and the diaphragm to detect free fluid between the liver and right kidney.
  ✓ Abdomen left upper quadrant (LUQ): examination of the splenorenal angle and diaphragm to detect free fluid between the spleen and left kidney.
  ✓ Subxiphoid-pericardial area (SUPH): examination of the pericardial space to detect pericardial effusion.
  ✓ Suprapubic view (BLADDER): examination of the pelvis to detect free fluid in the bladder and Douglas space [20].

### 2.4. Training of the Nurse Who Collected the Data

Data collection was performed by a nurse with 11 years of clinical experience, 7 of which were in the ED. The nurse was a postgraduate student in Advanced Emergency and Intensive Care Nursing and had completed a structured training program in POCUS and FAST, which included theoretical instruction, successful written examinations and supervised clinical training. The total duration of the training was 81 h, 20 of which were online training, including 6 months of clinical training under the supervision of a specialist physician.

During the training period, the nurse-performed FAST examinations as part of supervised clinical practice. However, a predefined minimum number of supervised examinations, formal competency thresholds, and standardized assessment criteria were not specified as part of the study protocol. In addition, no formal assessment of interobserver agreement was conducted prior to study initiation beyond the completion of the training and supervised clinical practice.

### 2.5. Description of the Sample Collection and FAST Procedure

#### 2.5.1. Procedure for Inclusion and Performance of the Examination

All ED physicians were informed about the study and agreed to the data collection procedure. For each injured person who met the inclusion criteria, the ED physicians issued a referral note for the FAST to be performed, which included the patient’s unique admission number.

The nurse then approached the patient (or their relatives, if the patient was unable to communicate), provided relevant information and offered participation in the study. After consent was given, the patient was enrolled in the study with a coded number to ensure anonymity.

The nurse-performed the FAST according to the predefined four-window protocol, with a total duration not exceeding 5 min. A positive FAST was defined as the presence of free fluid in any of the four ultrasound windows.

#### 2.5.2. Ensuring Independence and Blinding

After the nurse completed the FAST examination, the findings were immediately recorded on a form marked ‘N’ (Nurse) and handed to the on-call physician at the ED, who kept them in a locked area. The ultrasound images were stored in the nurse’s secure electronic environment, without access by the second examiner.

The patient then underwent a FAST examination by a radiologist. FAST examinations were performed by ten radiologists as part of routine clinical practice. Although all were qualified specialists, their level of experience in FAST was not formally standardized or recorded. The radiologist was aware that a prior FAST examination had been performed but did not have access to the nurse’s findings or images. Similarly, the nurse was not informed of the radiologist’s findings. The radiologist did not have access to the results of other imaging tests (e.g., X-ray or CT) at the time of the FAST examination and was only aware of the type of injury.

The radiologist’s findings were recorded on the referral form issued by the ED physician and returned to the ED physician on duty. A copy of this form, marked ‘R’ (Radiologist), was kept in the same secure area. Only ED physicians acting as independent data collectors had access to this locked area.

These procedures were implemented to promote independent recording of findings and to support blinding by minimizing access to prior results between examiners. FAST examinations were performed sequentially as part of routine clinical practice; however, given the rapid nature of the examination, the time interval between assessments was expected to be short, although it was not systematically recorded or formally analyzed.

#### 2.5.3. Management of Severely Injured Patients and CT Utilization

In patients requiring Trauma Team activation, the nurse-performed the FAST examination in the presence of the Trauma Team’s surgeons. In these cases, no second examination was performed by a radiologist. Instead, patients were transferred directly for CT imaging of the abdomen and lower chest, in accordance with routine clinical practice.

CT findings were used as the reference standard for evaluating the diagnostic accuracy of the nurse-performed FAST. CT images were interpreted independently by the radiologist without access to the recorded FAST findings, as part of routine clinical practice. The CT findings were collected and recorded by the ED physician.

CT imaging was also performed in selected patients outside Trauma Team activation, based on clinical judgment and standard trauma management protocols, and was not systematically applied to all patients.

#### 2.5.4. Collection of Demographic and Clinical Data

Patient gender, age and type of injury were recorded from medical records. The study was completed on 31 December 2023, and the findings were independently compared by two ED physicians who were not involved in the ultrasound examinations.

FAST was performed following referral by the attending ED physician and did not affect patient clinical management. Ultrasound findings recorded for study purposes were not made available to the trauma or surgical teams for decision-making, and patient management was based on standard clinical assessment and imaging pathways. The examination was rapid, non-invasive, and did not delay the implementation of other diagnostic or therapeutic interventions, ensuring that ED operations were not disrupted.

### 2.6. Ethical Issues

The study was approved by the Cyprus National Bioethics Committee (Ref. No.: 2022.01.135) and was conducted in accordance with the principles of the Declaration of Helsinki. Written informed consent was obtained prior to study participation from all participants or, where patients were unable to communicate, from their legally authorized representatives. The consent form was available in Greek and English.

To ensure data protection, the patient’s name was replaced with a number. This number was unique for each patient and stored in the database. Access to the patient database and ultrasound images was restricted to the nurse-researcher and the study’s scientific coordinator.

This study was not prospectively registered in a clinical trial registry.

### 2.7. Statistical Analysis

Quantitative variables are presented as mean and standard deviation, while categorical variables are presented as absolute (n) and relative (%) frequencies.

Inter-rater agreement between the nurse and the physician for the assessment of the presence or absence of free fluid was evaluated using Cohen’s kappa (κ) for each ultrasound region (RUQ, LUQ, SUPH, BLADDER). In cases of lack of variability (i.e., when all findings belonged to the same category), kappa could not be calculated. Given the low prevalence of positive findings, kappa estimates were interpreted with caution, as agreement measures may be affected by limited variability and prevalence effects.

The diagnostic accuracy of nurse-performed FAST was assessed by comparing the findings with CT, which was used as the reference standard. Sensitivity, specificity, and positive and negative predictive values were calculated, along with their corresponding 95% confidence intervals (CI).

The two-sided level of statistical significance was set at 0.05. Statistical analysis was performed using IBM SPSS 21.0 (Statistical Package for Social Sciences) software.

## 3. Results

### 3.1. Patient Characteristics

During the study period, a total of 76 injured patients were assessed for participation in the study. Of these, 8 were excluded because they did not meet the inclusion criteria or declined participation. The final study sample consisted of 68 patients, whose demographic and clinical characteristics are presented in Table 1. Of these, 58 patients underwent FAST performed by both the nurse and the radiologists, and constituted the sample for the inter-rater agreement analysis.

Of these 58 patients, 23 also underwent CT, based on clinical indications and physician judgment, in accordance with routine trauma management protocols. In addition, 10 patients requiring Trauma Team activation underwent FAST performed only by the nurse, followed by CT, without a second examination by a radiologist, in accordance with clinical practice.

**Figure 1:**
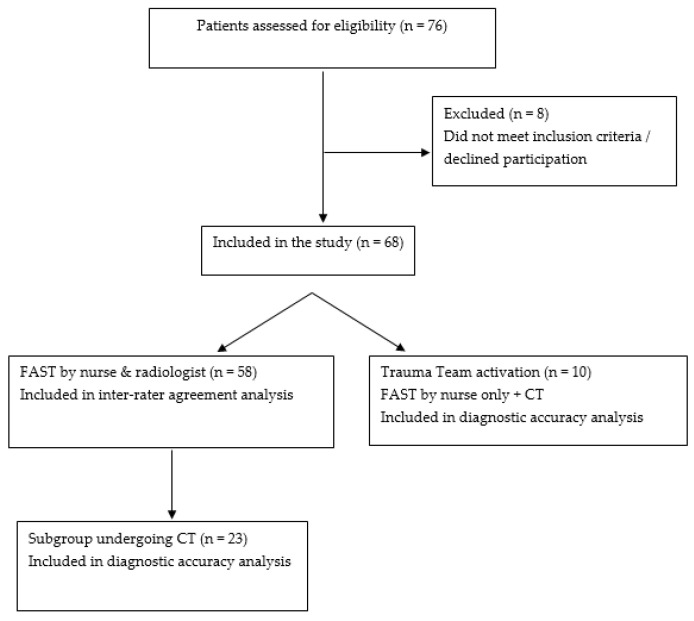
Flow diagram of patient inclusion and analysis.

In total, 33 patients underwent CT (23 from the agreement subgroup and 10 from the Trauma Team subgroup) and were included in the diagnostic accuracy analysis of nurse-performed FAST (Figure 1).

### 3.2. Overall Distribution of Findings by Region and Examiner (N = 58)

The distribution of the presence or absence of free fluid by anatomical region and examiner is presented in Table 2. In the RUQ region, fluid was detected in 3 patients (5.2%) by the nurse and in 4 patients (6.9%) by the radiologist. In the LUQ region, the nurse reported no positive findings, while the radiologist detected fluid in one patient (1.7%). In the SUPH region, both examiners identified fluid in one patient (1.7%). Finally, in the BLADDER region, no positive findings were recorded by either examiner. Overall, positive findings were limited in all anatomical regions.

### 3.3. Inter-Rater Agreement (N = 58)

A total of 58 patients underwent FAST performed by both the nurse and the radiologist and were included in the inter-rater agreement analysis. Agreement between examiners was assessed using kappa for the presence or absence of free fluid across four anatomical regions: RUQ, LUQ, SUPH and BLADDER.

Inter-rater agreement by anatomical region is presented below.

#### 3.3.1. Inter-Rater Agreement for the RUQ Region (N = 58)

The results of inter-rater agreement for the RUQ region are presented in Table 3. Of the 58 patients who underwent FAST by both examiners, agreement was observed in 57 cases (98.3%). Specifically, both examiners recorded the absence of fluid in 54 patients (93.1%) and the presence of fluid in 3 patients (5.2%). In one case (1.7%), disagreement was observed, with the nurse recording the absence of fluid and the radiologist recording the presence of fluid.

Kappa for the RUQ region was 0.85 (*p* < 0.001). However, this estimate was based on a small number of positive cases (n = 4), with only one discordant result, and should therefore be interpreted with caution.

#### 3.3.2. Inter-Rater Agreement for the LUQ Region (N = 58)

Agreement for the LUQ region is presented in Table 4. Of the 58 patients, observed agreement for absence of fluid was 98.3% (57 cases). In one case (1.7%), the radiologist recorded the presence of fluid, while the nurse recorded its absence. No cases were recorded in which the nurse reported the presence of fluid.

Due to the lack of variability (i.e., absence of positive findings by the nurse), kappa could not be reliably estimated for the LUQ region.

#### 3.3.3. Inter-Rater Agreement for the SUPH Region (N = 58)

Inter-rater agreement for the SUPH region is presented in Table 5. Of the 58 patients, agreement between the two examiners was observed in all cases (100%). Specifically, both examiners recorded the absence of fluid in 57 patients (98.3%) and the presence of fluid in one patient (1.7%), with no cases of disagreement. Kappa was 1.00 (*p* < 0.001), indicating complete agreement between the examiners, although this finding was based on a single positive case and should therefore be interpreted with caution.

#### 3.3.4. Inter-Rater Agreement for the BLADDER Region (N = 58)

Inter-rater agreement for the BLADDER region is presented in Table 6. Both examiners recorded absence of fluid in all 58 patients (100%), with no cases of fluid presence or disagreement.

Due to the lack of variability (i.e., all findings belonged to the same category), kappa could not be reliably estimated for the BLADDER region.

Overall, while observed agreement was high across regions, these estimates should be interpreted with caution, as the very low prevalence of positive findings and the small number of events limit the stability and interpretability of agreement measures.

### 3.4. Verification of Diagnostic Accuracy of Nurse-Performed FAST

In 23 patients, FAST was performed by both the nurse and the radiologists, and CT was also performed. These patients were included in the subgroup of 58 patients for whom the inter-rater agreement was assessed. In this subgroup, the diagnostic accuracy of nurse-performed FAST was evaluated using CT as the reference standard. Sensitivity, specificity, positive predictive value, and negative predictive value were calculated (Table 7).

Sensitivity was 100% (95% CI 15.81–100%), specificity was 100% (95% CI 83.89–100%), positive predictive value was 100% (95% CI 15.81–100%), and negative predictive value was 100% (95% CI 83.89–100%).

However, these estimates are based on a very small number of positive cases and are associated with wide confidence intervals.

In 10 additional patients requiring Trauma Team activation, FAST was performed only by the nurse in the presence of the Trauma Team’s surgeons, followed by CT. In this subgroup, the diagnostic accuracy of nurse-performed FAST was also assessed (Table 8). Sensitivity was 100% (95% CI 15.81–100%), specificity was 100% (95% CI 63.1–100%), positive predictive value was 100% (95% CI 15.81–100%) and negative predictive value was 100% (95% CI 69.15–100%).

However, these estimates are based on a very small number of positive cases and should be interpreted with caution.

## 4. Discussion

This study investigated the agreement between a nurse with relevant training and radiologists in performing FAST, as well as the diagnostic accuracy of the examination when performed by a nurse compared to CT, which was used as the reference standard.

However, these findings should be interpreted with caution, as the study was conducted in a single-center setting and included a single trained nurse, a limited sample size, a relatively short study duration, and a low number of positive cases. As such, the results should not be generalized beyond the study setting.

### 4.1. Agreement Between Examiners

The results of the study showed high agreement between the nurse and multiple radiologists in detecting free fluid in the RUQ region (κ = 0.85, *p* < 0.001). In the SUPH region, agreement was observed in all cases (κ = 1.00, *p* < 0.001). High observed agreement was also noted in the LUQ and BLADDER regions; however, kappa could not be reliably estimated due to lack of variability in the data.

These findings suggest a high level of agreement between the nurse and the radiologists when performing FAST; however, this should be interpreted with caution given the small number of positive cases and the study design. In addition, the findings are based on a single trained nurse in a single-center setting and may therefore reflect individual operator performance rather than the broader feasibility of nurse-performed FAST as a clinical practice model.

There is limited data in the literature regarding nurse-performed FAST and its comparison with physician assessments. The results of this pilot study suggest that, following appropriate training, an emergency care nurse may be able to perform FAST and interpret the findings with a high level of agreement; however, these results should not be generalized beyond the specific study setting.

This is particularly relevant in the ED settings, where the availability of radiologists may be limited relative to patient volume, potentially leading to delays in ultrasound assessment of trauma patients [21]. In this context, training nurses in FAST may potentially support more timely patient assessment and improved ED functionality. This enhances both the safe, effective and quality care of patients [22] and the advanced role of nurses in the emergency care setting [23,24,25]. However, these aspects were not directly evaluated in the present study and warrant further investigation.

### 4.2. Diagnostic Accuracy of Nurse-Performed FAST

The diagnostic accuracy of nurse-performed FAST was evaluated using CT as the reference standard. In the present study, sensitivity, specificity, and predictive values were estimated at 100%. However, these estimates should be interpreted with extreme caution, as they were derived from a very small number of patients and an extremely limited number of positive cases, resulting in wide confidence intervals and low precision. Therefore, these findings should be considered preliminary and not indicative of true diagnostic performance.

Compared with the limited available literature [17,18], the diagnostic estimates observed in the present study are higher and less stable. More specifically, in the study by Bowra et al. [17], nurses with experience in trauma management were trained in FAST through a one-day ultrasound seminar, which included three hours of theoretical training and three hours of practical training. They then participated in a certification process that involved performing and recording at least 25 FAST examinations. In this study, nurses performed FAST on 242 patients with suspected torso injuries in cases where a physician certified in performing FAST was not immediately available. The results were compared with CT findings or surgical findings obtained during hospitalization. Nurse-performed FAST demonstrated a sensitivity of 84.4%, specificity of 98.4%, a positive predictive value of 94.2% and a negative predictive value of 95.3% for the detection of free intra-abdominal fluid, with an overall diagnostic accuracy of 95.0%.

Henderson et al. [18] evaluated the ability of five nurse practitioners to perform ultrasound examinations after 16 h of training in POCUS (eight hours of theoretical training and eight hours of practical training), as well as one year of clinical experience under the guidance of emergency physicians in the ED. A total of 227 ultrasound examinations were recorded in this study, 25 of which were FAST examinations for trauma patients with suspected abdominal injury. Although no specific sensitivity, specificity or predictive value indices are reported for FAST examinations, the overall adequacy of the images obtained by the nurses was 86%, suggesting that the majority of ultrasound images were considered adequate for clinical evaluation. The quality and interpretation of the images were evaluated by an experienced emergency physician with expertise in ultrasound imaging. Overall, evidence from these studies suggests that nurse-performed FAST may be feasible following appropriate training; however, the magnitude and consistency of diagnostic performance vary across settings and study designs. The discrepancies observed compared with prior literature are likely attributable to the small sample size and the low prevalence of positive cases, rather than reflecting true differences in performance.

In the present study, training consisted of 81 h of theoretical and practical instruction, including 20 h delivered online, as well as 6 months of supervised clinical practice. Previous research suggests that online training may support the development of basic ultrasound skills; for example, one study [26] reported that inexperienced medical students were able to acquire fundamental practical ultrasound skills following online instruction.

The importance of ultrasound training is consistently emphasized in studies involving nurses working in the ED [17,18,27]. However, training programs vary substantially in duration and structure, limiting comparability across studies.

The existing literature emphasizes the importance of both theoretical and practical training in developing POCUS skills [22,28]. In medical education, ultrasound training is often introduced as early as undergraduate programs [29] and continues throughout specialization [30]. In contrast, the integration of ultrasound into nursing education remains limited. Although some postgraduate nursing programs may include ultrasound training, evidence of its incorporation into nursing curricula is scarce. Despite not being a standard component of undergraduate or postgraduate nursing education, its importance is increasingly recognized in specific areas of clinical practice.

In practice, training in POCUS and FAST is often delivered through continuing professional development in clinical settings [31,32,33,34,35,36], although these programs are not standardized. Further research is needed to define optimal training pathways and competency benchmarks for nurses performing FAST in emergency care settings. Although the nurse in the present study underwent extensive training, the study design does not allow conclusions to be drawn regarding the impact of training intensity on diagnostic accuracy.

### 4.3. Limitations

It is important to acknowledge certain limitations of the present study. First, this was a pilot study conducted at a single center, which limits the generalizability of the findings. Second, the sample size was relatively small.

Third, the relatively short study duration may have limited patient recruitment and the number of positive cases, potentially affecting the representativeness of the findings.

Fourth, a convenience sample was used, as patient inclusion depended on the availability of the participating nurse; therefore, not all eligible patients may have been included during the study period, introducing potential selection bias.

Fifth, the study did not systematically record the number or characteristics of eligible patients who were not included, and no comparison was performed between included and non-included cases; therefore, selection bias cannot be excluded.

Sixth, patient inclusion was also limited to specific time periods during which the trained nurse was available; therefore the included time windows may not fully represent routine ED operations across all shifts.

Seventh, the eligibility criteria, including the requirement for informed consent, the exclusion of life-threatening cases, and the exclusion of specific patient groups (e.g., patients with language barriers, BMI > 40, or pregnancy), may have led to the underrepresentation of more critically ill or complex patients, thereby introducing potential selection and spectrum bias.

Eighth, FAST examination was performed by a single nurse, which may limit the generalizability of the results. The findings may also reflect individual operator performance rather than the broader feasibility of nurse-performed FAST as a clinical practice model. In addition, FAST examinations were performed by multiple radiologists as part of routine clinical practice, and their level of experience was not formally standardized, which may have introduced inter-operator variability.

Ninth, the training process was not based on predefined competency thresholds or standardized assessment criteria, and interobserver proficiency was not formally evaluated prior to study initiation. In addition, the quality of ultrasound images was not formally assessed by an independent expert, and therefore the technical adequacy of the examinations could not be evaluated.

Tenth, complete blinding between examiners was not feasible, as FAST examinations were performed sequentially and the second examiner was aware that a prior examination had been conducted. The time interval between assessments and the duration of FAST examinations were not systematically recorded or analyzed, and potential clinical changes were not systematically recorded. These factors may have introduced a degree of shared clinical context and may have influenced the observed inter-rater agreement.

Eleventh, CT imaging was not performed systematically in all patients but was based on clinical indications and routine trauma management protocols. As a result, different diagnostic pathways were followed (i.e., nurse-performed FAST with radiologist assessment and CT in some cases, and nurse-performed FAST with CT in Trauma Team activation cases). This approach may have introduced partial verification bias, as diagnostic accuracy estimates were derived from a selected subgroup of patients who underwent CT and may not fully represent the entire study population.

Twelfth, the number of patients included in the diagnostic accuracy analysis was limited, and the number of positive cases was particularly small. This may affect the estimation of sensitivity and specificity and lead to wide confidence intervals. In addition, the very low prevalence of positive findings may have influenced the observed inter-rater agreement, as high agreement may partly reflect concordance in negative classifications rather than consistent identification of positive cases, thereby limiting the stability and interpretability of agreement measures.

Finally, no formal a priori sample size calculation was performed for either the inter-rater agreement or the diagnostic accuracy analyses, as this study was designed as a pilot study. Therefore, the sample size may not be sufficient to provide precise or stable estimates, particularly for diagnostic performance.

Overall, these factors may limit the generalizability of the findings, and the results should therefore be interpreted with caution.

## 5. Conclusions

In conclusion, this pilot study suggests that a trained emergency care nurse may achieve a high level of agreement with physicians when performing and interpreting FAST examinations in trauma patients presenting to the emergency department.

With regard to the secondary objective, the findings provide preliminary insights into the diagnostic accuracy of nurse-performed FAST compared with CT. However, further investigation is required to more reliably assess diagnostic performance.

Overall, these results support the feasibility of nurse-performed FAST in emergency care settings. Further studies involving larger samples, multiple operators, multicenter designs, and standardized training protocols are needed to validate these findings, better define their role in clinical practice, evaluate their integration into routine care, and investigate their potential impact on patient safety.

## Figures and Tables

**Table 1:** Demographic and clinical characteristics of patients (N = 68).

Variable	Value
Age (years)	
Mean (Standard Deviation)	49.3 (23.1)
Minimum–Maximum	19–93
Gender, n (%)	
Men	41 (60.3)
Women	27 (39.7)
Age groups, n (%)	
<40	28 (41.2)
40–60	16 (23.5)
>60	24 (35.3)
Type of injury, n (%)	
Traffic accidents	40 (58.8)
Falls	25 (36.8)
Beatings	3 (4.4)

**Table 2:** Distribution of findings by anatomical region according to the radiologist’s and nurse’s assessment (N = 58). Data are presented as n (% of total).

Anatomical Region	Absence of Fluid (Radiologist)	Absence of Fluid (Nurse)	Presence of Fluid (Radiologist)	Presence of Fluid (Nurse)
RUQ	54 (93.1)	55 (94.8)	4 (6.9)	3 (5.2)
LUQ	57 (98.3)	58 (100)	1 (1.7)	0 (0)
SUPH	57 (98.3)	57 (98.3)	1 (1.7)	1 (1.7)
BLADDER	58 (100)	58 (100)	0 (0)	0 (0)

**Table 3:** Inter-rater agreement for FAST in the RUQ region (N = 58). Data are presented as n (% of total).

Examiner	Radiologist: Absence of Fluid	Radiologist: Presence of Fluid	Total
Nurse: Absence of fluid	54 (93.1)	1 (1.7)	55 (94.8)
Nurse: Presence of fluid	0 (0)	3 (5.2)	3 (5.2)
Total	54 (93.1)	4 (6.9)	58 (100)

**Table 4:** Inter-rater agreement for FAST in the LUQ region (N = 58). Data are presented as n (% of total).

Examiner	Radiologist: Absence of Fluid	Radiologist: Presence of Fluid	Total
Nurse: Absence of fluid	57 (98.3)	1 (1.7)	58 (100)
Nurse: Presence of fluid	0 (0)	0 (0)	0 (0)
Total	57 (98.3)	1 (1.7)	58 (100)

**Table 5:** Inter-rater agreement for FAST in the SUPH region (N = 58). Data are presented as n (% of total).

Examiner	Radiologist: Absence of Fluid	Radiologist: Presence of Fluid	Total
Nurse: Absence of fluid	57 (98.3)	0 (0)	57 (98.3)
Nurse: Presence of fluid	0 (0)	1 (1.7)	1 (1.7)
Total	57 (98.3)	1 (1.7)	58 (100)

**Table 6:** Inter-rater agreement for FAST in the BLADDER region (N = 58). Data are presented as n (% of total).

Examiner	Radiologist: Absence of Fluid	Radiologist: Presence of Fluid	Total
Nurse: Absence of fluid	58 (100)	0 (0)	58 (100)
Nurse: Presence of fluid	0 (0)	0 (0)	0 (0)
Total	58 (100)	0 (0)	58 (100)

**Table 7:** Diagnostic accuracy of nurse-performed FAST compared with computed tomography (reference standard) (N = 23). Data are presented as n.

Examiner		CT Findings		Total
		Absence of Fluid	Presence of Fluid	
Nurse	Absence of fluid	21	0	21
	Presence of fluid	0	2	2
	Total	21	2	23

**Table 8:** Diagnostic accuracy of nurse-performed FAST compared with computed tomography (reference standard) in Trauma Team activation cases (N = 10). Data are presented as n.

Examiner		CT Findings		Total
		Absence of Fluid	Presence of Fluid	
Nurse	Absence of fluid	8	0	8
	Presence of fluid	0	2	2
	Total	8	2	10

## Data Availability

Full data are available upon reasonable request due to ethical and privacy restrictions.

## Abbreviations

The following abbreviations are used in this manuscript:

CT	Computed Tomography
ED	Emergency Department
FAST	Focused Assessment with Sonography in Trauma
LUQ	Left Upper Quadrant
POCUS	Point Of Care Ultrasound
RUQ	Right Upper Quadrant
SUPH	Subxiphoid-pericardial area
